# Positive Outcomes of Long-Term Relationship Satisfaction Trajectories in Stable Romantic Couples: A 10-Year Longitudinal Study

**DOI:** 10.1007/s41042-024-00201-1

**Published:** 2024-12-02

**Authors:** Michelle Roth, Selina A. Landolt, Fridtjof W. Nussbeck, Katharina Weitkamp, Guy Bodenmann

**Affiliations:** 1 Department of Psychology, Clinical Psychology for Children/Adolescents and Couples/Families, University of Zurich, Zurich, Switzerland; 2 Department of Psychology, Methods for Intensive Data in Psychology, University of Konstanz, Konstanz, Germany

**Keywords:** Romantic couples, Relationship satisfaction, Affect, Mental health, Life satisfaction, Latent class growth analysis, Longitudinal, Dyadic

## Abstract

Growing evidence challenges the longstanding assumption of a universal decline in relationship satisfaction for all couples by demonstrating variability in the change. However, knowledge about how differential long-term trajectories of relationship satisfaction are associated with positive individual functioning is lacking. Using dyadic latent class growth analysis, we studied long-term trajectories of relationship satisfaction of *N* = 300 mixed-gender couples over 10 years and examined positive outcomes in latent subgroups. At the final assessment, partners reported on positive outcomes, specifically affect, mental health, and life satisfaction. Depending on their subgroup membership, couples systematically differed: Couples in the subgroup with high initial and relatively stable relationship satisfaction reported the most favorable outcomes—more positive affect, better mental health, and higher life satisfaction—as compared to subgroups with declining and/or lower relationship satisfaction. The findings show the importance of considering the variability in long-term relationship satisfaction trajectories and point to their relevance for positive functioning.

**Supplementary Information:**

The online version contains supplementary material available at 10.1007/s41042-024-00201-1.

## Introduction

Past research has demonstrated the vital role the satisfaction of romantic relationships plays for couple stability (Karney & Bradbury, 1995), individual psychological (Holt-Lunstad et al., 2008), and physical health (Robles et al., 2014), as well as for well-being (Proulx et al., 2007). Yet, the focus was primarily on negative outcomes of low relationship satisfaction (Roberson et al., 2018), such as depressive symptoms or dissolution of the couple in the long-term, for example. Less is known about favorable outcomes of long-term happy relationships: Do high-quality relationships enable people to flourish? Can happy romantic relationships contribute to human functioning in the long term, and if so, how? To further the understanding of these questions, an in-depth examination of relationship development is especially important in long-term committed romantic relationships as effects of both, positive and negative, experiences accumulate over time (Umberson et al., 2006).

Given the importance of relationship satisfaction, it is crucial to understand how it changes over time. A recent meta-analysis (Bühler et al., 2021) integrated cross-sectional and longitudinal findings on the development of relationship satisfaction across the life span. Thereby, cross-sectional results comparing relationship satisfaction at different ages indicated a decrease in relationship satisfaction from age 20 reaching the lowest point at age 40 before it increased again until age 65 when it plateaued. Integrating cross-sectional findings on the metric of relationship duration indicated a cyclic pattern of relationship satisfaction with decreases within the first ten years of the relationship, increases until 20 years, whereafter it decreases again (Bühler et al., 2021). However, besides this normative developmental perspective, longitudinal information on stable relationships is crucial to understand the experiences within a given couple over time. Hereby, the meta-analytic results showed relationship satisfaction to decline over time within a given relationship with the strongest declines in the early years of a relationship (Bühler et al., 2021). Yet, there is evidence that the trajectories are more complex in nature, and not all couples follow this average decline: In a review by Proulx et al. (2017) on studies applying group-based approaches, different subgroups of change in relationship satisfaction over time were identified. Furthermore, Karney and Bradbury (2020) even stated that the majority of couples stay relatively satisfied over long periods of time. The integrated results in a review by Proulx et al. (2017) point to either stability or decline in relationship satisfaction with minimal evidence for increasing relationship satisfaction over time (Proulx et al., 2017).

Linking this variability in the course of relationship satisfaction to later positive outcomes might reveal important findings and contribute to a more differentiated understanding of the role of changes in relationship satisfaction in the long-term for positive functioning. However, while several studies were linking longitudinal group-based approaches in studying relationship satisfaction to negative outcomes such as divorce (see Proulx et al., 2017 for a review) to our knowledge, no study has examined positive outcomes. While Kamp Dush et al. (2008) studied a positive outcome, namely life satisfaction, of subgroup trajectories, and identified differences between subgroups, these subgroups did not specifically relate to relationship satisfaction in a narrower sense – whereby "relationship satisfaction is the subjective evaluation of one's relationship." (Keizer, 2014, p. 5437) –but rather also encompassed constructs such as sexual satisfaction. Hence, while these are valuable initial findings, it remains open to explore how variability in the change of relationship satisfaction is linked to positive outcomes.

### Linking Relationship Satisfaction and Outcomes

How, on a theoretical level, does relationship satisfaction impact a person in the long term? Different lines of reasoning and theoretical frameworks exist, aiming to explain the link between the quality of the relationship and a person’s health and well-being: A general model was proposed by Slatcher and Selcuk (2017), describing the processes of how close relationship features influence health outcomes. Both marital strains (negative aspects of the relationship) and marital strengths (positive aspects of the relationship such as high satisfaction) impact psychological mechanisms and ultimately physical health. In the marital discord model (Beach et al., 1990) it is assumed that dissatisfaction with the relationship leads to increased stress and negative interactions between spouses such as hostility, verbal and physical aggression, criticism, and blame, and decreased positive experiences such as cohesion, intimacy, and support all of which affects well-being. Accordingly, in a satisfying relationship, the negative characteristics should be less pronounced, and more cohesion, intimacy, and support should be experienced. Regarding the latter, its beneficial effects are explained by two mechanisms: (1) The main-effect model assumes a positive effect of social support irrespective of the stress level, and (2) the stress-buffering model assumes that support buffers stress and thus protects a person from its harmful effects (Cohen & Wills, 1985). Indeed, the process of how partners communicate their stress to each other, provide mutual support, and jointly cope with stress (i.e., dyadic coping) as conceptualized within the Systemic-Transactional Model (Bodenmann, 1997, 2005) – has been repeatedly linked to better individual and couple well-being (e.g., Landolt et al., 2023; Roth et al., 2022; Weitkamp et al., 2021). An additional line of reasoning for the link between relationship satisfaction and a person’s functioning builds upon the broaden-and-build theory (Fredrickson, 1998, 2001, 2004). The theory states that the experience of positive emotions ‘(i) broadens people’s attention and thinking; (ii) undoes lingering negative emotional arousal; (iii) fuels psychological resilience; (iv) builds consequential personal resources; (v) triggers upward spirals towards greater well-being in the future; and (vi) seeds human flourishing.’ (Fredrickson, 2004, p. 1375). Although these models do not directly refer to long-term experiences in close relationships, we can build on them and argue that frequently experienced positive interactions in satisfied close relationships (see Algoe, 2019) should lead to positive outcomes in the long-term.

### Positive Outcomes of Relationship Satisfaction

Meta-analytic evidence supports the assumption of the above-mentioned theoretical models about relationship satisfaction being relevant to a person’s well-being (Proulx et al., 2007), physical health (Robles et al., 2014), and psychological health (e.g., Goldfarb & Trudel, 2019). Since the current study aims to contribute to the understanding of the relevance of relationship satisfaction trajectories for positive individual functioning, we will focus on affect and life satisfaction, which have been proposed as the defining components of subjective well-being (Diener et al., 1999), and on mental health. Below, previous research on how these constructs relate to relationship satisfaction is outlined.

The impact of relationship satisfaction on affect is often studied in the form of positive and negative affect as an element of subjective well-being as defined by Diener et al. (1999). Relationship satisfaction is positively associated with positive affect and negatively associated with negative affect (e.g., Londero-Santos et al., 2021; Love & Holder, 2016). Satisfaction with life as the other element of subjective well-being (Diener et al., 1999) was found to be concurrently and prospectively associated with relationship satisfaction in previous research: Cross-sectionally, higher relationship satisfaction was found to be associated with higher satisfaction with life (Holt-Lunstad et al., 2008; Londero-Santos et al., 2021). Longitudinally, an increase in relationship satisfaction was associated with an increase in life satisfaction (Hilpert et al., 2016; Stanley et al., 2012), and higher relationship satisfaction predicted a positive change in life satisfaction (Gustavson et al., 2016).

An association between relationship satisfaction and mental health is well-documented in previous research. Higher relationship satisfaction was found to be associated with better mental health (Holt-Lunstad et al., 2008; Proulx et al., 2007; Whisman, 1999). Furthermore, the integrated cross-sectional evidence was described to be robust in showing that higher relationship satisfaction is associated with lower symptoms of depression (Goldfarb & Trudel, 2019). Furthermore, in multiple studies, higher relationship satisfaction was shown to predict lower depressive symptoms longitudinally (e. g., Beach & O’Leary, 1993; Beach et al., 2003) with a causal direction between higher relationship satisfaction and subsequent better mental health in women (Downward et al., 2022). Braithwaite and Holt-Lunstad (2017) concluded that a broad range of mental health outcomes are influenced by a person’s romantic relationship. In a recent review, Whisman et al. (2021) furthermore concluded that "the existing body of research evidence supports the claim that relationship distress is a causal risk factor for depression." (p. 233). It seems therefore conceivable that high relationship satisfaction as opposed to relationship distress serves as a protective factor against mental health problems.

Nevertheless, conflicting or inconsistent findings to the ones outlined above exist as well. In a review, Goldfarb & Trudel, 2019, described the evidence for the longitudinal pathway between relationship satisfaction and mental health as less conclusive than for cross-sectional associations between those variables. For example, studies applying models that capture bidirectional associations over time found that relationship satisfaction and depressive symptoms reciprocally influence each other over time (Gustavson et al., 2012; Whisman & Uebelacker, 2009). Similar findings exist for life satisfaction whereby relationship satisfaction not only impacted life satisfaction prospectively, but life satisfaction also influenced relationship satisfaction prospectively (Be et al., 2013; Gustavson et al., 2016).

### Current Study

Previous research has shown that different relationship satisfaction trajectory subgroups exist (Proulx et al., 2017) and these should therefore be taken into account when studying long-term effects. Whereas previous studies have mostly focused on newlywed couples (e.g., Lavner & Bradbury, 2010), long-term stable romantic couples might be especially important to consider since the effects of relationship satisfaction might accumulate over time. Furthermore, relationship satisfaction was shown to be relevant for positive outcomes such as well-being (e.g., Proulx et al., 2007) life satisfaction (e.g., Gustavson et al., 2016). However, studies examining the relation between positive outcomes and differential trajectories (i.e., subgroups with specific trajectories) of relationship satisfaction are missing. We, hence, aim to gain a deeper understanding of the long-term effects of variability in relationship satisfaction over time to address these currently existing gaps in the literature.

In the first step, we therefore applied a dyadic group-based approach to investigate differential development of relationship satisfaction in romantic couples over ten years. We expected to find several subgroups of change in relationship satisfaction with stability or varying degrees of decline. Yet, we had no assumption about the number of groups, nor did we presume partners within a couple to show the same pattern of development over time. After having identified subgroups with different trajectories of relationship satisfaction over time, we aimed to study differences between these subgroup trajectories in positive outcomes. Specifically, we examined affect, life satisfaction, and mental health. Thereby, we hypothesized to find the most favorable outcomes in couples with high and relatively stable relationship satisfaction over time compared to couples with lower relationship satisfaction and/or steeper declines over time. However, we had no assumption about whether a subgroup with high initial but strongly declining or a subgroup with a lower but slightly increasing level of relationship satisfaction would fare better or worse regarding positive outcomes. More favorable outcomes would mean more positive affect, higher life satisfaction, and better mental health.

## Methods

### Transparency and Openness

The current study was not preregistered and the data are not publicly accessible. The data are not publicly available due to their containing information that could compromise the privacy of research participants. However, data can be made available from the corresponding author upon reasonable request. Information on the codebook and material, the MPlus code as well as more details about the whole longitudinal research project are accessible in the supplemental material.

### Participants and Procedure

For the current study, data from a 10-year prospective study (2011–2021) were used where we followed 368 mixed-gender couples (*n* = 736 individuals) in annual assessments (T1–T10). Couples were recruited via newspaper and radio announcements. The following inclusion criteria were used: participants must have been in their current relationship for at least a year, be fluent in German, and be 18 years or older. The study was approved by the Ethics Committee of the University of Zurich (No. 2013.1.1, No. 17.8.2; No. 19.8.13; No. 20.6.18**)** and all couples gave their written informed consent. Participants filled in questionnaires on a wide range of individual and dyadic variables at each time point (see https://doi.org/10.15139/S3/IUGVBK for more information on the study). We used measures of relationship satisfaction from all ten occasions of measurement to identify potential latent subgroups with differential trajectories. Measures on affect, life satisfaction, and mental health at the last occasion of measurement were used as outcomes of latent subgroups. Couples that separated during the study (*n* = 68) were excluded since the focus of the current paper was on stable romantic couples and missing data of separating couples cannot be assumed to be missing at random. The final analytic sample of the current study was *N* = 300 mixed-gender couples (*n* = 600 individuals) at baseline (T1). Throughout the study, the attrition rate was 51% with 189 couples dropping out. The number of drop out at each time point were as follows: T2: 39; T3: 41; T4: 35; T5: 18; T6: T7: 10; T8: 12; T9: 9; T10: 8.

At T1, the average age was *M* = 51.1 (*SD* = 17.80) years for women and *M* = 53.0 (*SD* = 17.7) years for men. Relationship duration was *M* = 25.1 years (*SD* = 18.3 years) on average and 75% (*n* = 224) of couples were married at T1. A majority of couples (71% of men and 72% of women) reported having at least one child at T1. Participants were mostly Swiss (96% among women, 95% among men) or German (6% of women, 8% of men). The sample had middle to higher socioeconomic backgrounds with a median income of 120‘000 Swiss Francs per couple (which is approximately 140‘000 US Dollars or 128'000 Euros) and was highly educated, with 29% of women and 51% of men holding a university degree.

### Measures

#### Relationship Satisfaction

We used the short version of the German Couple Satisfaction Index (CSI-4; Funk & Rogge, 2007) to assess relationship satisfaction over time. Four global statements about one’s relationship are rated on a 6-point Likert scale (ranging from 1–6) by each participant. Items have different anchors, one example is 1 = *not at all* to 6 = *completely*. Higher values indicate higher relationship satisfaction. An example item is the following: ‘In general, how satisfied are you with your relationship?’. At all time points, reliability ranged from good to excellent in women (α_T1_ = 0.83; α_T2_ = 0.84; α_T3_ = 0.89; α_T4_ = 0.89; α_T5_ = 0.89; α_T6_ = 0.92; α_T7_ = 0.92; α_T8_ = 0.93; α_T9_ = 0.92; α_T10_ = 0.88;) and from good to excellent in men (α_T1_ = 0.89; α_T2_ = 0.88; α_T3_ = 0.91; α_T4_ = 0.84; α_T5_ = 0.86; α_T6_ = 0.91; α_T7_ = 0.91; α_T8_ = 0.91; α_T9_ = 0.92; α_T10_ = 0.90;)

#### Affect

To measure affect, we used the short version of the Multidimensional Mood Questionnaire (MDMQ; Steyer et al., 1994) at the last occasion of measurement*.* The short version of the MDMQ consists of 12 items assessing three dimensions of mood (pleasant-unpleasant; awake-sleepy; calm-restless). In this study, we only used the pleasant-unpleasant dimension (4 items). For each item participants have to indicate how they feel with respect to a specific adjective (e.g., “Do you feel good?”) on a 5-point scale ranging from 1 = *not at all* to 5 = *very much so.* Higher values indicate a more positive mood. Reliability was excellent in the current study (α = 0.94 women; α = 0.93 men).

#### Life Satisfaction

We used one global item (‘Overall, are you satisfied with your life?’) to assess life satisfaction at the last occasion of measurement. Participants rated this item on a 5-point scale ranging from 1 = *not at all* to 5 = *very,* with higher values indicating higher life satisfaction.

#### Mental Health

As an indicator of mental health, we used the German version of the General Health Questionnaire (GHQ-12; Goldberg, 1992). The scale consists of 12 items assessing psychological distress (e.g., ‘Have you recently felt constantly under strain?’). Items are answered on a 4-point scale with regard to one’s usual condition (e.g., 1 = *much less than usual* to 4 = *more so than usual*). In the original scale, higher values indicate more severe psychological distress. For the current study, we reverse-coded the answers during the data analysis so that higher values indicate less psychological distress, i.e., better mental health. In the current study, reliability was excellent α = 0.93 for women and good α = 0.85 for men.

### Data Analysis

To identify subgroups in the trajectory of relationship satisfaction over time, we used Latent Class Growth Analyses (LCGA). Compared to a traditional latent growth curve model, where average intercepts and slopes are estimated for the whole sample, group-based approaches such as LCGA allow for the identification of subgroups with distinct growth factors (Nagin, 1999). As recommended by Jung and Wickrama (2008), we started our analyses by estimating traditional and latent class growth curve models for men and women separately to avoid computational burden at an initial stage of model building and to obtain an overview of potential patterns in the data. To test for potential non-linear trajectories, we ran models with freely estimated loading parameters and models with quadratic slope factors for men and women respectively. In the final step, we proceeded with a dyadic LCGA. In a dyadic model, any combination of different types of trajectories found for the two partners separately is principally possible. As recommended by Herle et al. (2020), we used the following goodness-of-fit criteria to identify the optimal number of classes: the Akaike Information Criterion (AIC), Bayesian Information Criterion (BIC), and the sample-size corrected version of the BIC (c-BIC), where smaller values indicate a better fit. Furthermore, we used the parametric bootstrap likelihood ratio test (BLRT) to determine significant differences between the models as recommended in the literature (Nylund et al., 2007). Additionally, we took other considerations into account, such as successful convergence, high entropy (values closer to 1 indicating good classification), and high posterior probabilities (Jung & Wickrama, 2008) as well as interpretability and avoidance of too small classes (Herle et al., 2020), since the above-mentioned goodness-of-fit criteria do not necessarily agree in the choice of the best model (Herle et al., 2020). After identifying the optimal number of latent classes, we included relationship duration as a control variable in the model. Finally, we explored subgroup differences in affect, life satisfaction, and mental health, assessed at T10, as outcomes of the latent classes. To do so, we used the BCH method (named after Bolck et al., 2004) to assess mean differences between classes in both partners' reports of the outcomes. For missing data, we did not have to reject the assumption of missing at random in the final analytic sample (*N* = 300) as indicated by the Little Test (Little, 1988) (χ^2^(446) = 449.95, *p* = 0.439 for women and χ^2^(496) = 499.92, *p* = 0.442 for men); hence, we handled missing data using full information maximum likelihood (see Enders, 2011 for more information on the handling of missing data). Data preparation, descriptive statistics, and measurement invariance testing were conducted in RStudio version 2023.06.1 + 524 (R Core Team, 2023). The dyadic LCGA including the BCH method was estimated in Mplus Version 8.7 (Muthén & Muthén, 1998–2017).

## Results

In Table 1, means, standard deviations, and correlations of study variables are presented. Overall, the sample was satisfied and exhibited relatively high levels of positive affect, mental health, and life satisfaction.

**Table 1:** Means, standard deviations, and correlations of study variables

Variable		Women	Men	1	2	3	4	5	6	7	8	9	10	11	12	13
		*M* (*SD*)	*M* (*SD*)													
1	RS T1	5.20 (0.67)	5.20 (0.69)	.56*	.67*	.71*	.71*	.67*	.69*	.59*	.63*	.60*	.49*	.28*	.39*	.16*
2	RS T2	5.10 (0.74)	5.20 (0.66)	.77*	.53*	.70*	.73*	.71*	.65*	.66*	.58*	.58*	.50*	.29*	.46*	.17*
3	RS T3	5.20 (0.77)	5.20 (0.76)	.73*	.71*	.60*	.75*	.67*	.64*	.52*	.62*	.61*	.47*	.29*	.44*	.15
4	RS T4	5.10 (0.80)	5.20 (0.67)	.70*	.66*	.75*	.56*	.77*	.72*	.65*	.72*	.65*	.58*	.33*	.50*	.21*
5	RS T5	5.10 (0.86)	5.20 (0.73)	.67*	.64*	.73*	.75*	.56*	.75*	.70*	.69*	.71*	.56*	.25*	.42*	.16*
6	RS T6	5.10 (0.80)	5.10 (0.71)	.62*	.57*	.72*	.73*	.74*	.55*	.78*	.77*	.75*	.60*	.38*	.51*	.26*
7	RS T7	5.00 (0.85)	5.10 (0.75)	.63*	.62*	.69*	.71*	.73*	.79*	.57*	.77*	.70*	.54*	.32*	.46*	.25*
8	RS T8	5.00 (0.85)	5.00 (0.79)	.49*	.50*	.62*	.66*	.70*	.73*	.75*	.60*	.78*	.62*	.32*	.48*	.22*
9	RS T9	5.00 (0.87)	5.00 (0.80)	.55*	.58*	.64*	.67*	.66*	.73*	.76*	.74*	.51*	.68*	.37*	.54*	.24*
10	RS T10	4.90 (0.91)	5.10 (0.74)	.47*	.48*	.51*	.57*	.57*	.59*	.63*	.68*	.71*	.59*	.46*	.67*	.34*
11	Affect T10	3.71 (0.94)	3.93 (0.81)	.13	.10	.22*	.16*	.20*	.22*	.18*	.26*	.19*	.29*	.17*	.67*	.81*
12	Life satisfaction T10	4.15 (0.85)	4.22 (0.72)	.29*	.27*	.37*	.26*	.30*	.36*	.34*	.38*	.32*	.38*	.62*	.36*	.54*
13	Mental health T10	3.05 (0.54)	3.13 (0.50)	.06	.02	.08	.04	.10	.09	.09	.15	.11	.23*	.75*	.56*	**.07**

Values below the diagonal are for men, those above the diagonal for women; values on the diagonal in bold are between-partner correlations; *RS* Relationship Satisfaction, *T1-T10* Measurement time points; * p < .05

### Subgroups of Relationship Satisfaction Trajectories

As described in the data analysis section, we started by setting up models for each gender separately and tested for free, linear, and quadratic slopes. Yet, models with freely estimated loadings for the slope parameters did not converge and the inclusion of a quadratic slope factors did not improve model fit, hence we continued with linear LCGA and tested two to four classes in each gender separately. Due to too small class size, we did not consider models with more than four classes (a portion of the smallest class was below 10%). Fit indices for these models are listed in Table S1 in the supplemental material. Next, we combined the two genders in a dyadic model. Again, we found that estimating models with more than four classes resulted in very small classes (less than 10%). The final model was a three-class model with slope variances constrained to zero due to estimation issues. We did not impose constraints across classes, on the intercept variance or on the covariance between the men's and women's intercepts. Fit indices for the dyadic models are listed in Table S2 in the supplemental material. Running a Monte-Carlo-analysis (10,000 runs), we explored the power to detect the three sub-classes in a potential population showing the same properties as our sample. We found that the three classes could be reliably discovered with a minimum average latent class probability of 0.923. To control for relationship duration, class membership was regressed on relationship duration in the final model. One couple had missing values in relationship duration and was therefore automatically excluded by Mplus from subsequent analyses.

**Fig. 1:**
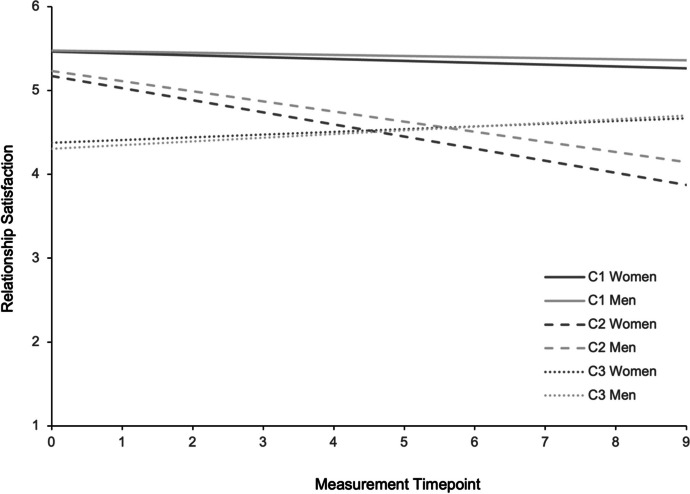
Relationship satisfaction trajectories of latent classes over 10 years. Note. C1 = First latent class, i.e., high relatively stable class; C2 = Second latent class, i.e., high declining class; C3 = Third latent class, i.e., low increasing class

Parameter estimates and fit indices for the final model with three classes are listed in Table 2 and Fig. 1 shows the relationship satisfaction trajectories of the three latent classes over 10 years. The largest class (C1) consisted of 65% (*n* = 194) of the couples. Compared to the two other classes, couples in this class (C1) showed the highest level of initial relationship satisfaction. Women in this class showed a small significant negative slope with an absolute decrease in relationship satisfaction over the ten years of 0.22 units in the scale of relationship satisfaction. The negative slope in men was not significant indicating stability in relationship satisfaction over ten years. We refer to this class as a *high relatively stable* class where *high* refers to the intercept and relatively *stable* refers to the slope (which is stable in men and slightly decreasing in women). The second largest class (C2) consisted of 19% (*n* = 56) of the couples. Both partners in this class also reported a relatively high initial level of relationship satisfaction but both men and women showed significant and relatively large negative slopes: Women decreased by 1.44 units and men by 1.21 units in relationship satisfaction over 10 years. We refer to this class as the *high declining* class where *high* refers to the intercept and *declining* refers to the slope. In both C1 and C2, the slopes indicated a higher absolute decrease in relationship satisfaction for women compared to men. The third class (C3) consisted of 17% (*n* = 50) of the couples. Compared to the two other classes, couples in C3 reported the lowest level of initial relationship satisfaction. Men and women in C3 showed a slight increase in their relationship satisfaction as indicated by small but significant positive slopes resulting in an absolute increase of 0.32 units in women and 0.44 units in men over 10 years. Thus, this class was termed the *low increasing* class where *low* refers to the intercept and *increasing* refers to the slope. In the current sample, all couples remained above the cut-off of the CSI for relationship distress (Funk & Rogge, 2007). The results of the logistic regression analysis of the control variable relationship duration on group membership revealed that higher relationship duration significantly increased the probability of belonging to C1 or C3 as compared to C2.

**Table 2:** Parameter estimates of the dyadic latent class growth analysis with three latent classes

Subgroup	*n* (%)	Intercept (Variance)	Slope^a^
C1: High relatively stable	194 (65%)		
Women		5.463*** (0.125***)	−0.022***
Men		5.475*** (0.114***)	−0.013
C2: High declining	56 (19%)		
Women		5.170*** (0.414***)	−0.144***
Men		5.232*** (0.261***)	−0.121***
C3: Low increasing	50 (17%)		
Women		4.378*** (0.551***)	0.032*
Men		4.304*** (0.382**)	0.044**

^a^ = Slope variance was constrained to be zero. *n* = number of dyads. Aikaike Information Criterion (AIC) = 6011.683. Bayesian Information Criterion (BIC) = 6178.203. Sample size corrected BIC (c-BIC) = 6035.49. Bootstrapped Likelihood Ration Test *p*-value = .000. Entropy of the Model = .73. * p < .05. ** p < .01. *** p < .001

### Outcomes of Relationship Satisfaction Subgroup Trajectories

**Fig. 2:**
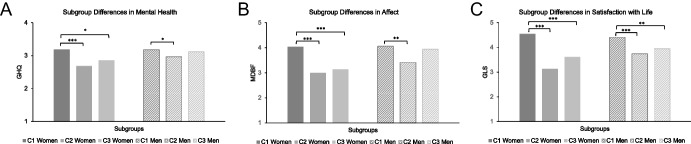
Differences between relationship trajectory classes in affect, life satisfaction, and mental health. Note. C1 = First latent class, i.e., high relatively stable class; C2 = Second latent class, i.e., high declining class; C3 = Third latent class, i.e., low increasing class. MDMQ = Multidimensional Mood State Questionnaire; GLS = General Life Satisfaction; GHQ = General Health Questionnaire. * p < .05. ** p < .01. *** p < .001

Results on the class differences between the three latent classes in each construct are depicted in Fig. 2 and Table 3.

**Table 3:** Differences between long-term relationship satisfaction trajectory classes in positive outcomes

	Women *M (SD)*				Men *M (SD)*			
Outcome	C1	C2	C3	χ^2^-Equality Tests (Cohen's *d* effect size for mean difference)	C1	C2	C3	χ^2^-Equality Tests (Cohen's *d* effect size for mean difference)
Affect	4.098 (0.094)	3.034 (0.193)	3.122 (0.200)	C1 vs. C2*** (1.15) C1 vs. C3*** (0.72) C2 vs. C3 (0.09)	4.083 (0.087)	3.513 (0.169)	3.934 (0.188)	C1 vs. C2** (0.67) C1 vs. C3 (0.12) C2 vs. C3 (0.45)
Life Satisfaction	4.629 (0.070)	3.158 (0.190)	3.603 (0.211)	C1 vs. C2*** (2.13) C1 vs. C3*** (0.81) C2 vs. C3 (0.42)	4.454 (0.075)	3.783 (0.132)	3.935 (0.183)	C1 vs. C2*** (0.91) C1 vs. C3** (0.44) C2 vs. C3 (0.17)
Mental Health	3.227 (0.053)	2.670 (0.122)	2.857 (0.140)	C1 vs. C2*** (1.07) C1 vs. C3* (0.42) C2 vs. C3 (0.27)	3.200 (0.054)	2.97 (0.085)	3.112 (0.151)	C1 vs. C2* (0.43) C1 vs. C3 (0.10) C2 vs. C3 (0.43)

C1 = First latent class, i.e., high relatively stable class; C2 = Second latent class, i.e., high declining class; C3 = Third latent class, i.e., low increasing class. χ^2^ = Chi-Square. * p < .05. ** p < .01. *** p < .001

### Affect

Descriptively, women in the *high relatively stable* class (C1) reported the highest amount of positive affect, followed by the *low increasing* class (C3) again followed by the *high declining* class (C2). Significant differences were found between C1 and C2 as well as between C1 and C3, such that women in the *high relatively stable* class reported significantly more positive affect than the *high declining* and the *low increasing* class (C1 > C2, C3), while the latter two classes did not differ in their reported affect. The significant differences were medium to large in terms of effect sizes. In men, the same descriptive pattern was found as in women. However, in men, only the difference between the *high relatively stable* and the *high declining* class was significant with a medium effect size, while the *high relatively stable* and the *low increasing* class did not differ significantly. See Fig. 2A for a visual representation of the results.

### Life Satisfaction

Descriptively, the highest level of life satisfaction was reported in the *high relatively stable* class, followed by the *low increasing* class followed by the *high declining* class in both women and men. Both men and women in the *high relatively stable* class reported the highest life satisfaction among the three classes (C1 > C2, C3) with large effect sizes in women and small to large effect sizes in men. Neither men nor women differed significantly in their life satisfaction between the *high declining* and the *low increasing* class, indicating equal levels of life satisfaction in those two classes. See Fig. 2B for a visual representation of the results.

### Mental Health

Descriptively, women and men in the *high relatively stable* class (C1) reported higher mental health scores, i,e, less psychological distress), followed by the *low increasing* class (C3) followed by the *high declining* class (C2). Significant differences were found in women such that the *high relatively stable* class reported the highest levels of mental health (C1 > C2, C3), whereas women in the *high declining* and the *low increasing* class did not significantly differ in their reported mental health scores. The significant differences were small to large in terms of effect size. Men’s mental health scores significantly differed between the *high relatively stable* class (C1) and the *high declining* class (C2) with a small effect size. No other subgroup comparison was significant. See Fig. 2C for a visual representation of the results.

## Discussion

In the current study, we identified three subgroups with different relationship satisfaction trajectories over 10 years. Furthermore, we examined positive outcomes of long-term relationship satisfaction trajectories. Specifically, we studied the effect of class membership on affect, life satisfaction, and mental health.

In line with our first hypothesis, we found several subgroups of change in relationship satisfaction over time. Specifically, we identified three classes of couples with distinct trajectories: 1) high relatively stable (65%), 2) high declining (19%), and 3) low increasing (17%) couples. This aligns with previous research that also identified different subgroup trajectories (Proulx et al., 2017) and with the view that most couples remain relatively stable for a long time (Karney & Bradbury, 2020). However, in contrast to our hypothesis, we identified one subgroup with slightly increasing relationship satisfaction over time. While integrated findings on studies using group-based approaches only found minimal evidence for increases in relationship satisfaction trajectories (Proulx et al., 2017), a more recent study also identified a subgroup with increasing relationship satisfaction in women (Kanter et al., 2021). Findings about increasing relationship satisfaction need to be further replicated and it remains to be understood under which conditions such a finding is made and what enables couples to increase their relationship satisfaction over time. In the current study, couples with longer relationship durations were more likely to belong to the low increasing class (or to the high relatively stable class). Meta-analytic results indicated largest declines in relationship satisfaction in the early years of a relationship (Bühler et al., 2021). In these earlier years couples potentially undergo more relationship transitions such as the birth of a child, which can cause stress (e.g., Roth et al., 2022) and impact relationship satisfaction (Mitnick et al., 2009). Couples at later stages of their relationship, however, have overcome these stressful times and couples might have more time and resources again to engage in positive relationship maintenance behaviors such as joint leisure activities, sharing gratitude, or engaging in humorous conversations that are known to be beneficial for the relationship (Ogolsky et al., 2017). The higher experienced stress in early stages of a relationship might also serve as one explanation for the following finding: While high initial relationship satisfaction was associated with stability over time for the majority couples (couples in the high relatively stable subgroup, C1), there is a considerable portion of participants that showed relatively high initial relationship satisfaction but large declines over time (C2). The key question is why these two classes (C1 and C2), both with high relationship satisfaction at the start of the study, show such distinct patterns over time? The mentioned higher levels of stress during these earlier relationship stages might be a potential explanation. Furthermore, examining interpersonal variables that are known to be relevant for relationship satisfaction such as dyadic coping (see Falconier et al., 2015 for a meta-analysis) – that is how couples communicate about stress, support each other in times of stress, and jointly cope with stressors (Bodenmann, 1995, 1997) – might be of relevance to further the understanding of the differentiated development of these two subgroups.

With regard to the outcomes of relationship satisfaction trajectory subgroups, overall and in line with our hypothesis, we found that couples in the high and relatively stable class reported more favorable outcomes than couples that initially started high but showed strong declines over time. Thus, partners who were satisfied with their relationship initially and stayed satisfied over ten years reported more positive affect, had better mental health, and were overall more satisfied with their lives. Thereby, the effect sizes were descriptively larger in women compared to men. Comparing couples starting and staying relatively highly satisfied to couples with lower but increasing relationship satisfaction clearly supported our hypothesis for women while less clear for men: Women in the high relatively stable satisfaction class reported higher positive affect, better mental health, and higher life satisfaction, compared to women in the low increasing subgroup, whereas in men, a significant difference between these two subgroups was only found for life satisfaction with a small effect size. Overall, these findings align with theoretical frameworks as well as previous research that showed higher relationship satisfaction to be related to more positive affect (e.g., Londero-Santos et al., 2021), better mental health (e.g., Proulx et al., 2007), and higher life satisfaction (e.g., Londero-Santos et al., 2021). However, we extend these findings by using not only a single-time point measure of relationship satisfaction or manifest change to predict future outcomes but by analyzing latent classes of change and by combining different positive outcomes in one study. Overall, the high relatively stable subgroup showed the most favorable outcomes after ten years, pointing to the relevance of a satisfying relationship for positive individual functioning.

More exploratory, we examined whether the high declining or the low increasing class showed more favorable outcomes. While in previous research the moderate initial satisfaction groups tended to fall between higher and lower trajectory classes regarding outcomes (Proulx et al., 2017), this was not the case in our study. Our ‘middle group’ i.e., the group in between high and low initial satisfaction, showed the least favorable outcomes descriptively. Various explanations are possible: First, it is difficult to directly compare results between studies, since different numbers of classes and different trajectory shapes are identified. Furthermore, with its steep decline, the high declining class exhibits the lowest relationship satisfaction after ten years. These couples underwent a deterioration – they initially had a satisfying relationship which worsened over time which impacted outcomes negatively. Thus, once the satisfaction declined strongly, the high initial satisfaction seems no longer to be a resource but rather makes the hurtful change more salient. This contrast might go along with deception and disillusionment and therefore impact well-being and life satisfaction. However, the comparisons between the high declining and the low increasing subgroup were all non-significant and the related effect sizes were small. Therefore, the results suggest that high and relatively stable relationship satisfaction is favorable for positive outcomes, and both a steeply declining and relatively lower relationship satisfaction, despite slight increases over time, are negatively associated with later positive outcomes.

In contrast to most previous studies using group-based approaches, we applied a dyadic model, which allows us to draw conclusions about the couple as a unit instead of identifying subgroups in both genders separately. Our results indicate that partners within one couple change relatively similarly over time, i.e., partners within each of the three subgroups showed similar initial levels and trajectories over time. However, men in the high relatively stable subgroup showed stability in their relationship satisfaction over 10 years whereas women reported slight declines. Alongside similarities within subgroups, we found more similarities than dissimilarities between women's and men's relationship satisfaction trajectories and the outcome variables. However, whereas women in the high relatively stable and the low increasing subgroup differed with regard to all three outcomes, men in these subgroups only differed with regard to life satisfaction. This raises the question of whether men’s affect and mental health are differentially affected by their relationship satisfaction than women’s affect and mental health. However, the effect of gender on the association between relationship satisfaction and mental health was studied in previous research with inconsistent findings (e.g., Beach et al., 2003; Kurdek, 2005; Roberson et al., 2018). While some studies found stronger associations between relationship quality and mental health for women (Dehle & Weiss, 1998) others reported no significant differences (Kurdek, 2005). The gender similarities hypothesis states that women and men are more similar than dissimilar in most psychological variables (Hyde, 2005) and also with regard to relationship satisfaction (Jackson et al., 2014; Johnson et al., 2022). Our results are largely in line with similarity between women and men and we thus refrain from overinterpreting minimal gender differences.

### Limitations and Future Research

In the current sample, socioeconomic status was relatively high, only mixed-gender couples were included, and the sample consisted of persons with a Western European background. Thus, the generalizability of the findings is limited and the lack of inclusivity, as stated by Randall et al. (2022), was also present in the current study. Clearly, more diverse samples regarding socioeconomic status, sexual orientation, and cultural background are needed. Furthermore, we used questionnaire data only. These are more prone to biases whereas behavioral observation of outcomes would allow for more objective measures, to capture real-time positive processes such as shared laughter or affectionate touch, and to observe phenomena such as the couple's climate. Another limitation regarding the measures is that mental health was assessed in terms of (the absence of) symptoms of psychological distress, while according to the World Health Organization (WHO) mental health encompasses more than the mere absence of distress (WHO, 1946). Measures that reflect a broader range of mental health indicators should therefore be used in future studies. Furthermore, while our results offer interesting findings about how relationship satisfaction trajectory subgroups differ in the long-term in relevant outcomes, drawing causal conclusions should be conducted with care since vice-versa effects from the used outcomes variables on relationship satisfaction are conceivable as well. Indeed, previous studies have found bidirectional effects between relationship satisfaction and different constructs (e.g., Braithwaite & Holt-Lunstad, 2017; Sağkal & Özdemir, 2020).

Important questions remain open and should be addressed in future research: First, the above discussed direction of effect remains to be further studied and bidirectional effects between relationship satisfaction and the constructs used in the current study should be further investigated. Second, longitudinal studies with shorter time gaps, such as weekly diary studies, are needed to examine these dynamics more closely since some more state-like variables might fluctuate in shorter time dimensions such as days or weeks (e.g., affect). With this in mind, the co-evolution of relationship satisfaction and positive outcomes could be studied, e.g., focusing on the question of whether two variables are changing in the same manner, a question also posed by (Proulx et al., 2007), or whether different rates and trajectories of change are conceivable (e.g., linear vs. another trajectory shape)? Third, change in relationship satisfaction per se might not have an effect on the outcomes unless a particular threshold of change, which might additionally be different depending on the initial level of relationship satisfaction, has been reached. More knowledge regarding such thresholds could be especially important for preventive programs and help to better identify couples at special risk.

## Conclusion

In sum, the results of the current study point to the importance of considering variability in the change of relationship satisfaction and show the relevance of relationship satisfaction trajectories for positive individual functioning. While the literature seems to be clearer about the link between relationship satisfaction trajectories and negative outcomes such as separation/divorce (Lavner & Bradbury, 2010), studies examining the link between relationship satisfaction trajectories and positive outcomes are lacking. The current study is thus, to our knowledge, the first one to combine dyadic latent subgroup analyses of relationship satisfaction with positive outcomes. In sum, the results show the importance of long-term relationship satisfaction trajectories for positive outcomes. Particularly, high and relatively stable relationship satisfaction is beneficial for affect, life satisfaction, and mental health. Thus, our results support the notion of the importance of a dyadic approach, not only for individual happiness as previously stated (Hilpert et al., 2016) but for a broader range of positive individual outcomes. Therefore, prevention and public health initiatives should implement an interpersonal approach and address romantic relationships when aiming to promote positive individual functioning.

## Supplementary Information

Below is the link to the electronic supplementary material.

Supplementary file1 (DOCX 41 kb)

## Data Availability

The current study was not preregistered, and the data are not publicly accessible. The data are not publicly available due to their containing information that could compromise the privacy of research participants. However, data can be made available from the corresponding author upon reasonable request. Information on the codebook and material, the MPlus code as well as more details about the whole longitudinal research project are accessible in the supplemental material.
