# Supplementary material for: Positive Outcomes of Long-Term Relationship Satisfaction Trajectories in Stable Romantic Couples: A 10-Year Longitudinal Study
Source: Int J Appl Posit Psychol. 2024 Dec 2;10(1):8. doi: 10.1007/s41042-024-00201-1 (PMC11611981; doi:10.1007/s41042-024-00201-1)
Supplement: Supplementary file 1 — Supplementary file1 (DOCX 41 kb) [file 41042_2024_201_MOESM1_ESM.docx]

**Supplemental Material**

| **Table S1** | | | | | | |
| --- | --- | --- | --- | --- | --- | --- |
| *Fit Indices for Model Comparison from a Two-Class to a Four-Class Model for Women and Men* | | | | | | |
| Statistic |  | Women |  |  | Men |  |
|  | 2 classes | 3 classes | 4 classes | 2 classes | 3 classes | 4 classes |
| AIC | 4002.998 | 3526.992 | 3436.270 | 3584.501 | 3243.525 | 3055.248 |
| BIC | 4002.998 | 3766.000 | 3514.049 | 3640.058 | 3310.193 | 3133.028 |
| c-BIC | 4010.984 | 3536.574 | 3447.450 | 3592.486 | 3253.108 | 3066.428 |
| PBLRT *p*-value | .0000 | .000 | .0000 | .000 | .000 | .000 |
| Entropy | .87 | .88 | .80 | .88 | .83 | .83 |
| Class size n (%) | 233 (78%),  67 (22%) | 111 (37%),  23 (8%),  166 (55%) | 167 (49%),  21 (7%),  42 (14%),  90 (30%) | 219 (73%),  81 (27%) | 110 (37%),  36 (12%),  154 (51%) | 132 (44%),  7 (2%),  53 (17%),  108 (36%) |
| *Note.* AIC = Aikaike Information Criterion; BIC = Bayesian Information Criterion; c-BIC = sample size corrected BIC; BLRT = Bootstrapped Likelihood Ratio Test. | | | | | | |

| **Table S2** |  |  |  |  |  |  |  |
| --- | --- | --- | --- | --- | --- | --- | --- |
| *Fit indices for Model Comparison for the Dyadic Latent Class Growth Model, from Two-Class to Four-Class Models* | | | | | | | |
| Statistic | 2-classes constrained LCGM | 3-classes constrained LCGM | 4-classes constrained LCGM | 2-classes  LCGM^a^ | 3-classes  LCGM^a^ | 4-classes  LCGM^a^ | Final 3-class model with control variable^b^ |
| AIC | 7922.017 | 7266.924 | 6975.493 | 6126.162 | 6028.128 | 5991.204 | 6011.683 |
| BIC | 7830.046 | 7392.853 | 7119.941 | 6255.794 | 6187.39 | 6180.097 | 6178.203 |
| c-BIC | 7922.017 | 7285.025 | 6996.256 | 6144.795 | 6051.02 | 6018.355 | 6035.49 |
| PBLRT *p*-value | .000 | .000 | .000 | .000 | .000 | .000 | .000 |
| Entropy | .93 | .87 | .85 | .65 | .70 | .72 | 0.73 |
| Class size *n* (%) | 62 (21%)  238 (79%) | 110 (37%)  162 (54%)  28 (9%) | 120 (40%)  16 (5%)  43 (14%)  120 (40%) | 69 (23%)  231 (77%) | 195 (65%)  57 (19%)  48 (16%) | 40 (14%)  177 (59%)  73 (24%)  10 (3%) | 50 (17%)  194 (65%)  56 (19%) |
| *Note.* LCGM = Latent Class Growth Model; AIC = Aikaike Information Criterion; BIC = Bayesian Information Criterion; c-BIC = sample size corrected BIC; BLRT = Bootstrapped Likelihood Ratio Test; ^a^ = no constraints across classes or on the intercept variance or the covariance covariance between the male and female partners intercepts, slope variance constrained to zero. ^b^ = corresponds to the 3-classes LCGM^a^ with relationship duration added as a control variable. | | | | | | | |

**Codebook Information and MPlus code**

The following **measures** were used:

- Relationship satisfaction: **German Version of the Couples Satisfaction Index (CSI-4)**

Funk, J. L. & Rogge, R. D. (2007). Testing the Ruler With Item Response Theory: Increasing Precision of Measurement for Relationship Satisfaction With the Couples Satisfaction Index. *Journal of Family Psychology, 21*(4), 572-583.

- Affect: **German short version of the Multidimensional Mood Questionnaire (MDBF**)

Steyer, R., Schwenkmezger, P., Notz, P., & Eid, M. (1994). Testtheoretische Analysen des Mehrdimensionalen Befindlichkeitsfragebogen (MDBF) [Theoretical analysis of a multidimensional mood questionnaire (MDBF)]. *Diagnostica, 40*(4), 320-328.

- Life Satisfaction: **One global item** (‘Overall, are you satisfied with your life?’)
- Mental Health: **German version of the General Health Questionnaire (GHQ-12)**

Goldberg, D. (1992). General health questionnaire (GHQ-12). *Windsor, UK: Nfer-Nelson*.

**Coding:**

The item codes follow a logical structure characterized by several components:

- Measurement time point: Represented by 'a' for the 1^st^, 'b' for the 2^nd^ measurement time point and so forth.
- Gender: Indicated by 'f' for female and 'm' for male.
- Acronym: Primarily used to signify the scale being employed.
- Sequential Item Numbering: Progresses continuously from 01 up to the largest assigned number.

For instance, the item code "afcsi04" deciphers as follows: It pertains to the 1^st^ measurement time point ('a'), is associated with the female gender ('f'), falls under the category of relationship satisfaction as evaluated by the CSI-4 scale, and corresponds to the 4^th^ item within this context.

Similarly, the scale codes also follow a structured pattern:

- Measurement time point: Represented by 'a' for the 1^st^, 'b' for the 2^nd^ measurement time point and so forth.
- Gender: Denoted by 'f' for female and 'm' for male.
- Acronym: Principally utilized to denote the specific scale
- In the event of a subscale being considered, an additional acronym signifies the subscale.

For instance, the acronym “afCSI” deciphers as follows: It pertains to the 1^st^ measurement time point ('a'), is associated with the female gender ('f') and is the scale value (CSI) of the CSI-4 scale.

| Variable | Measure | Acronym of scale in dataset | Acronym of subscales in dataset | Construction of scale value |
| --- | --- | --- | --- | --- |
| Relationship satisfaction | CSI-4 | CSI | No subscale | Relationship satisfaction is computed as the mean of the following items: csi01, csi02, csi03, csi04. Higher values indicate higher relationship satisfaction |
| Affect | MDBF | MDBF | gs | Positive-negative mood subscale (4 items) was used which is computed as the mean of the following items: mdbf01, mdbf05r, mdbf11, mdbf15r |
| Life Satisfaction | One item | gls | No subscale, item 01 |  |
| Mental Health | GHQ-12 | GHQ | No subscale | The mental health score is computed as the mean of the following items: ghq01, ghq02, ghq03, ghq04, ghq05, ghq06, ghq07, ghq08, ghq09, ghq10, ghq11, ghq12. Items were recoded such that higher values indicate better mental health. |

MPLUS Syntax for the final dyadic 3-Class Model with outcomes is depicted below:

*for the sake of readability not all variable names are depicted as it is an extensive dataset.

Title: LATENT CLASS GROWTH ANALYSIS (3 classes) predicting class membership

Data: FILE is finaldata.csv;

Variable: NAMES ARE

[extensive list of variable names*];

USEVARIABLES ARE

abez

jfgls01 jmgls01

afCSI bfCSI cfCSI dfCSI efCSI

ffCSI gfCSI hfCSI ifCSI

amCSI bmCSI cmCSI dmCSI emCSI

fmCSI gmCSI hmCSI imCSI

jfCSI jmCSI

jfMDBF_gs

jmMDBF_gs

jfGHQ jmGHQ;

MISSING ARE ALL (-99);

CLASSES = c(3);

AUXILIARY = jfgls01(bch) jmgls01(bch)

jfMDBF_gs(bch) jmMDBF_gs(bch)

jfGHQ(bch) jmGHQ(bch)

Analysis: type = MIXTURE missing;

STARTS = 500 20;

STITERATIONS = 20;

Model: %OVERALL%

if sf | afCSI@0 bfCSI@1 cfCSI@2 dfCSI@3 efCSI@4

ffCSI@5 gfCSI@6 hfCSI@7 ifCSI@8 jfCSI@9;

sf@0;!fixes within-class slope variances to zero.

im sm | amCSI@0 bmCSI@1 cmCSI@2 dmCSI@3 emCSI@4

fmCSI@5 gmCSI@6 hmCSI@7 imCSI@8 jmCSI@9;

sm@0; !fixes within-class slope variances to zero.

c on abez;

%c#1%

if;

im;

if with im;

%c#2%

if;

im;

if with im;

%c#3%

if;

im;

if with im;

Output: sampstat standardized

tech1 TECH8 TECH11 TECH14;

PLOT: SERIES = afCSI-jfCSI (sf) amCSI-jmCSI (sm);

TYPE = PLOT3;

More information on the longitudinal study can be publicly accessed at <https://doi.org/10.15139/S3/IUGVBK>
